# Molecular Simulation of SO_2_ Separation and Storage Using a Cryptophane-Based Porous Liquid

**DOI:** 10.3390/ijms25052718

**Published:** 2024-02-27

**Authors:** Pablo Collado, Manuel M. Piñeiro, Martín Pérez-Rodríguez

**Affiliations:** 1 Departamento de Física Aplicada, Universidade de Vigo, E36310 Vigo, Spain; pablo.collado@uvigo.es; 2 Instituto de Química Física Blas Cabrera, Consejo Superior de Investigaciones Científicas (CSIC), E28006 Madrid, Spain; martin.perez@iqf.csic.es

**Keywords:** porous liquid, SO_2_, cryptophane-111, radial distribution function, molecular dynamics

## Abstract

A theoretical molecular simulation study of the encapsulation of gaseous SO_2_ at different temperature conditions in a type II porous liquid is presented here. The system is composed of cage cryptophane-111 molecules that are dispersed in dichloromethane, and it is described using an atomistic modelling of molecular dynamics. Gaseous SO_2_ tended to almost fully occupy cryptophane-111 cavities throughout the simulation. Calculations were performed at 300 K and 283 K, and some insights into the different adsorption found in each case were obtained. Simulations with different system sizes were also studied. An experimental-like approach was also employed by inserting a SO_2_ bubble in the simulation box. Finally, an evaluation of the radial distribution function of cryptophane-111 and gaseous SO_2_ was also performed. From the results obtained, the feasibility of a renewable separation and storage method for SO_2_ using porous liquids is mentioned.

## 1. Introduction

Porous liquids (referred to as PLs hereafter for brevity) were first described by O’Reilly et al. [1] as fluid media with permanent porosity. This porosity is achieved though the suspension of large molecules, whose voids act as intrinsic permanent pores. This starting point opened a new path towards the development of innovative techniques in gas separation and storage. PLs combine the great variety and efficiency of the solid porous materials used for gas selective adsorption with the easier storage and handling nature of liquids at a large scale.

Porous liquids were firstly classified into three groups: types I, II, and III [2]. However, very recently, a new type, type IV, was proposed by Bennet et al. [3]. Type I PLs comprise liquids where each individual molecule presents a permanent and rigid cavity, so they cannot be auto-filled, thus making them hard to synthesise because of their need for this neat state. They present high melting points and a tendency to crystallise outside certain ideal range conditions [4]. The melting point can be reduced by reducing the porosity by adding flexible alkyl chains [5]. Type II PLs are composed of rigid cage molecules and a sterically hindered solvent that cannot enter cage cavities. The unnecessary presence of a neat state like in type I PLs, a solvent that presents high solubility with the cage molecule, and a larger size due to bigger pores make them easier to synthesise or simulate. Type III PLs consist of metal organic frameworks (MOFs) suspended in a sterically hindered solvent. The latter are easier to synthesise, and they eliminate the need for low melting points or high solubility because of being in suspension. However, they may present phase separation and generate precipitation due to the different nature of the pores and their distribution in the MOF, thus lowering their overall porosity. To avoid this, some studies have considered the use of nanoparticles or nanocrystals despite their big size and the consequent stability issues [6]. Finally, Type IV PLs are defined as MOF species with intrinsic porosity that is isolated in liquid form; this means that they do not need a solvent for their suspension. Few studies have so far been carried out on PLs and even less on gas absorption. There are some studies that suggest the possibility of developing a renewable route to adsorb CO_2_ under mild conditions [7,8,9,10,11,12,13]; similarly, in the study by Zou et al. [14], they used CO_2_ absorption as a form of storage for posterior catalysis usage. However, there is still limited knowledge on the adsorption of other greenhouse gases in porous liquids. We can cite, as an example, the work by Oltean et al. [15], where the CH_4_ interaction with cryptophane-111 (C-111) in the presence of H_2_ is described. Madhavi et al. [16] summarised the characteristics of the examples of different kinds of PLs that had been acquired experimentally.

**Figure 1:**
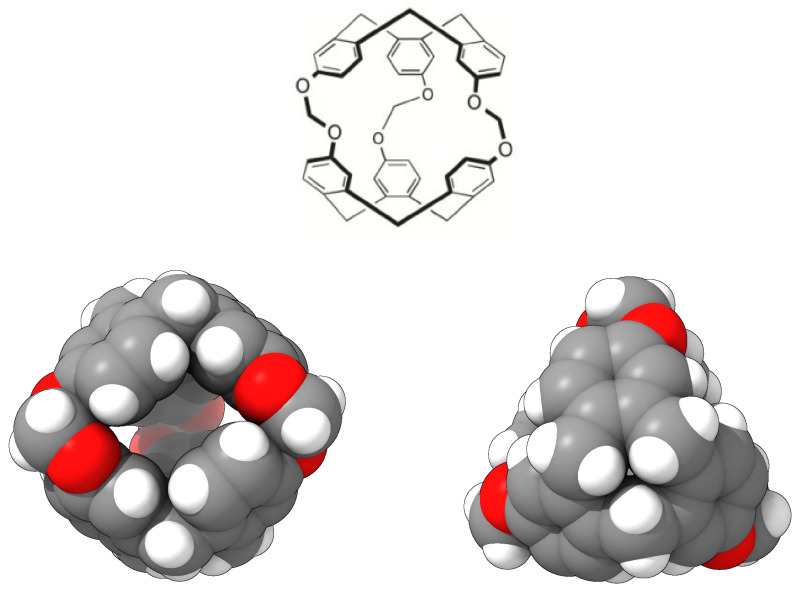
Chemical structure of cryptophane-111 (**top**) [22] and a three-dimensional model using van der Waals standard radii, as viewed from the side (**bottom left**) and top (**bottom right**) of the molecule. The side view shows the effective internal cavity and the three accesses to it: two at the back and one at the front. Carbon, hydrogen and oxygen atoms are colored in grey, white and red, respectively.

**Figure 2:**
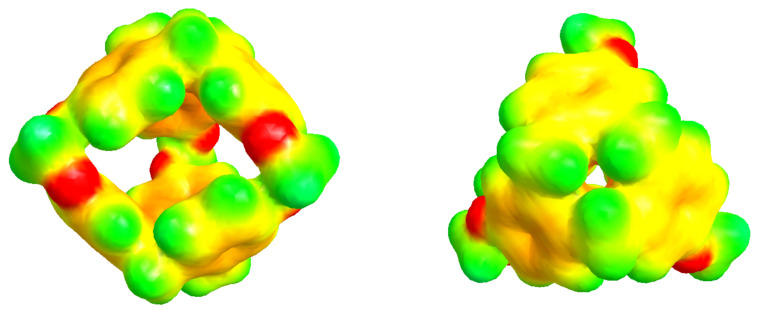
Representation of the electrostatic potential of a C-111 molecule, as viewed from the side (**left**) and top (**right**) of the molecule. Negative-potential regions are coloured in red, while positive-potential regions are coloured in green. The illustration was prepared by mapping electrostatic potential on the electronic density calculated by means of the AM1 semi-empirical method [23].

In this study, we analysed, using molecular simulations, the process of encapsulating SO_2_ in a porous liquid at different temperatures under laboratory conditions. We simulated a type II PL which is composed of the smallest molecule from the cryptophane family, namely, cryptophane-111 (C-111). In a previous work, we presented some results on CO_2_ and CH_4_ adsorption in a C-111 porous liquid [17]. This C-111 molecule was first analysed in this context by Fogarty et al. [18], and since then, that work has been intensively studied for its remarkable complexation with Xe [18,19,20]. Cryptophanes are aromatic molecules, and they are defined by three symmetric folds of cyclotribencylene in a crown conformation, which also defines an inner cavity with stable geometry. Because of their stereoisomerism, they present a great variety of structures and properties. The possibility of enclathrating a guest molecule in their cavity depends on the nature of their bonded moieties and their functional groups (as they define the size and flexibility of the cage geometry [21]). The narrow hollows of C-111 make it a suitable candidate molecule to study the encapsulation/separation of small gas molecules. We can observe the C-111 molecule in Figure 1. In Figure 2, we see a representation of the electrostatic potential of a C-111 molecule showing a negative potential inside the C-111 cavity.

SO_2_ was selected as the guest molecule in this study for several reasons. The first of them is the ecological and industrial interest in identifying a method to separate and capture this gas. Additionally, the ability of C-111 to encapsulate small molecules as Xe has been already reported in the literature. The Xe molecule has nearly the same volume as SO_2_, so in terms of cavity size, SO_2_ is a feasible candidate for this study. Finally, dichloromethane (DCM) was selected as solvent because it presents good solubility with both C-111 and SO_2_. The volume of the C-111 molecule inner cavity ranges from 32 Å^3^ to 72 Å^3^ in its most expanded state. Although DCM presents a molecular volume within that interval, it cannot get through the entrance of the cavity, as discussed by Buffeteau et al. [24]. SO_2_, however, is commonly presented commercially dissolved in DCM, so their mutual solubility does not represent a problem.

The choice of this particular type II porous liquid was motivated for various reasons. First of all, the adsorption performance demonstrated by C-111 in our previous work [17] is a positive reference. Additionally, we have experience in the application of molecular simulation methods to characterize gas diffusion through structured solid materials as hydrates or clathrates [25,26,27,28,29,30], and the process of gas adsorption in PLs presents many similar features to these inclusion solids. Moreover, the molecular simulation of type II PLs is computationally more accessible than other cases. For instance, type III PLs may experience precipitation, while type I present very complex molecular structures, and type IV show amorphous nature. All these features represent great limitations for a detailed description using molecular simulations [31], while the case selected in this study represents a good starting point for the future analysis of more complex scenarios. In summary, the proposed modelling strategy is a rigorous and feasible approach with the objective to evaluate a renewable method to separate and recover SO_2_. This study represents a contribution to the theoretical description of potential alternatives to greenhouse gas capture, with the aim to explore the capacity of PLs in this context.

## 2. Results and Discussion

### 2.1. Case A

**Figure 3:**
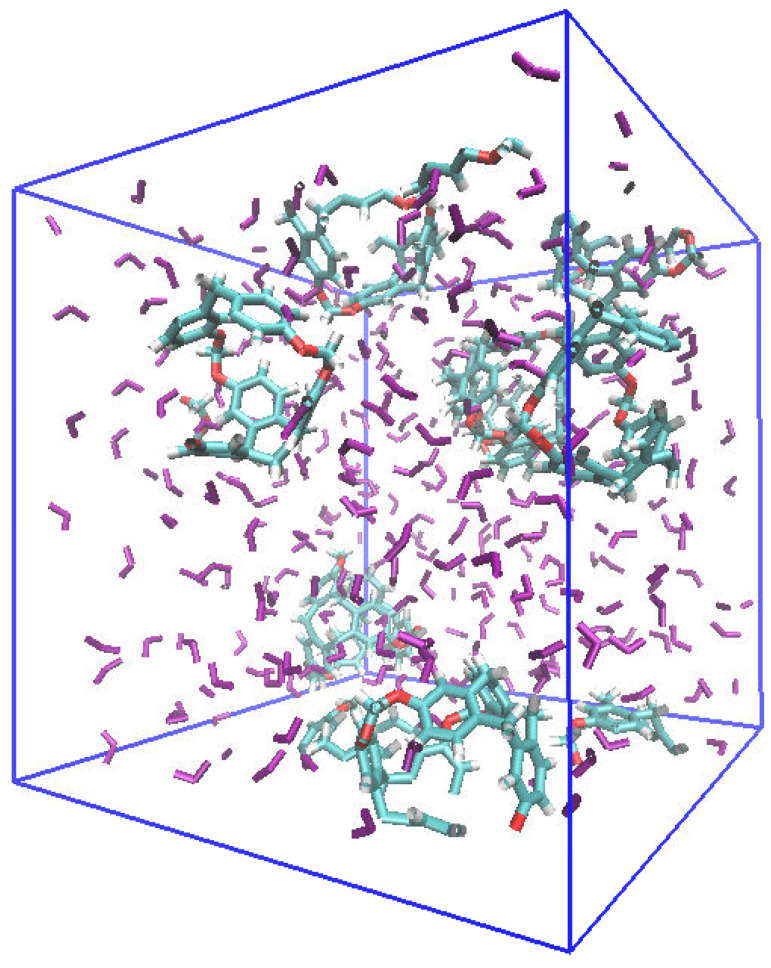
Snapshot of the (C-111 + DCM) porous liquid simulation box (case A) at the end of the simulation.

Two simulation boxes corresponding to the porous liquid (C-111 + DCM) and the binary mixture of the solvent with the captable molecule (DCM-SO_2_) were built, and run at temperatures of 283 and 300 K. The objective of this case was to identify the interactions between the molecular models used and determine the phase equilibria in each case. In the porous liquid (C-111 + DCM) simulation box, a mutual weak interaction among C-111 molecules was noticed, which resulted in short-lived agglomerations that did not finally achieve phase separation. Thus, this behaviour does not affect the C-111 capacity of absorption. The variation in the temperature in the range studied did not seem to affect this kind of weak interactions nor the mixing of the molecules. Figure 3 shows a snapshot of the simulation box, showing a homogeneous molecular distribution.

**Figure 4:**
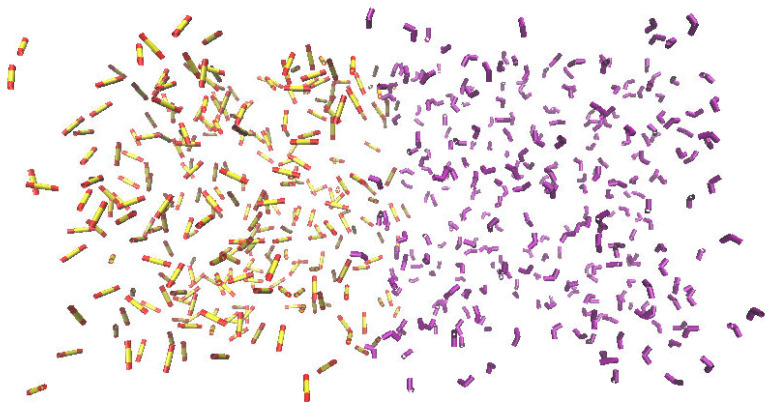

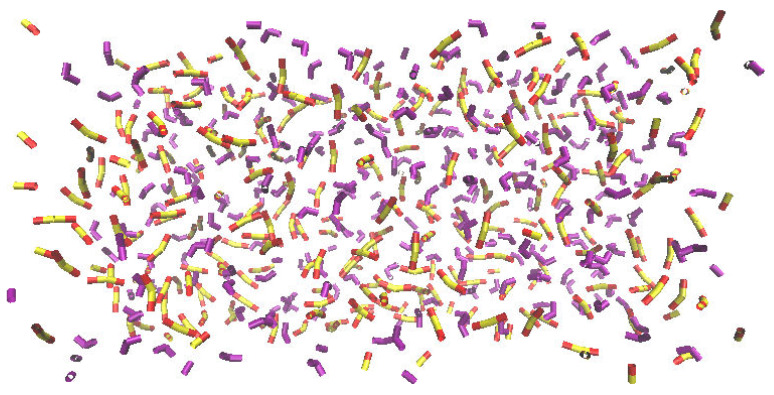
Initial (**top**) and final (**bottom**) snapshots of the simulated mixture of SO_2_ and DCM at 300 K. DCM molecules are depicted in purple.

For the simulation of the second case A simulation box, an initial biphasic layer was built with the objective to observe the mixing process of DCM and SO_2_. Complete miscibility was obtained at the end of the simulation under the tested thermodynamic conditions. As before, the temperature change did not affect the mixing of the two components. The initial and final states of this simulation box are shown in Figure 4.

### 2.2. Case B

Nine replica simulations for this system were performed at both 283 K and 300 K and 1 bar. For every replica, all molecules were inserted at random. All simulation runs were compared in terms of cavity occupation and time of occupation by selecting every single SO_2_ molecule manually and following its trajectory through the simulation. Table 1 lists C-111 occupation for every individual run and the average value for the complete set of runs performed. The standard deviations determined at 283 K and 300 K were very similar, but the occupation value was slightly higher at 283 K. The C-111 occupancy rate (OR) can be defined as the ratio between the number of SO_2_-occupied C-111 molecules at the end of the simulation, divided by the total number of C-111 molecules in the simulation box. The average values obtained for this initial test were 0.87 at 283 K and 0.84 at 300 K. This difference can be due to the slower diffusion of SO_2_ at the lower temperature, which might favour a correct orientation to access the C-111 pore. A faster occupation rate with time at 300 K can also be noticed. As before, this effect can be attributed to the thermal noise, as well as to faster absorption related to the temperature increase.

**Figure 5:**
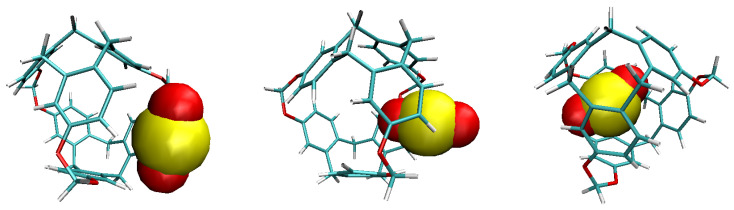
Sequence of SO_2_ molecule outside (**left**), entering (**centre**), and inside (**right**) the C-111 cavity.

The fact that there is a preferred insertion orientation can be also underlined. This is the case when the SO_2_ molecular axis is normal to the C-111 cavity entrance. Even though the C-111 cavity volume is larger than the SO_2_ molecular volume, its entrance is slightly smaller, making the SO_2_ being sterically hindered in any other orientation. A remarkable absorptive capacity of C-111 towards SO_2_ must also be noted, and this prevents the adsorbed molecule to get out of the cavity once it has entered. A sequence of the process of a SO_2_ molecule entering a C-111 cavity can be seen in Figure 5.

**Figure 6:**
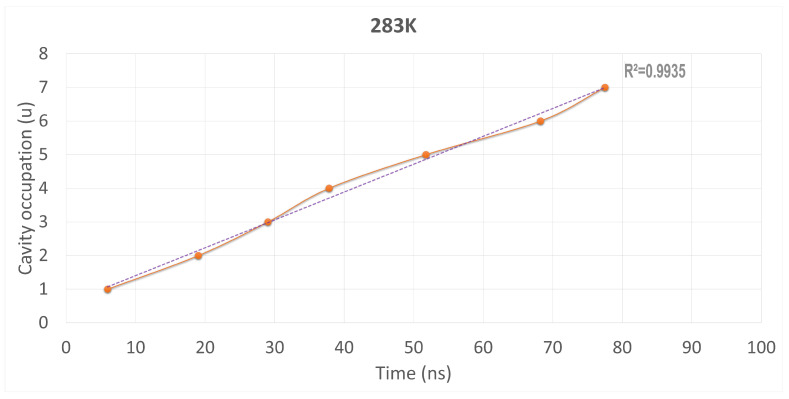

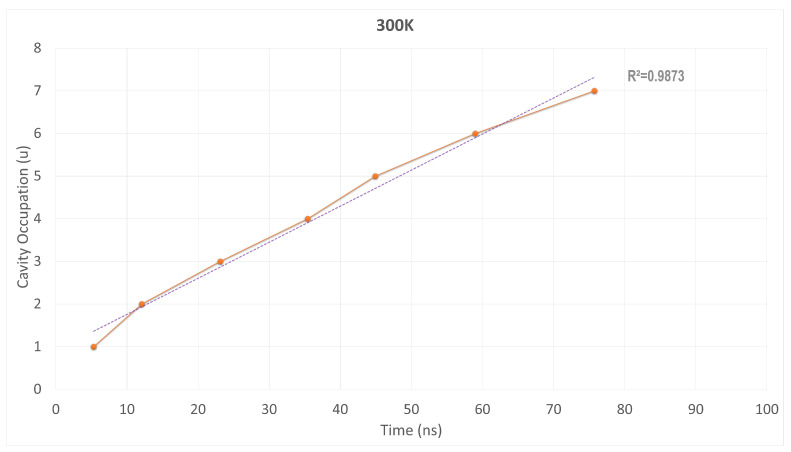
Cavity occupation trend plotted vs. simulation time, at both temperatures.

Figure 6 plots the absorption trend represented vs. simulation time scale. We can observe that this absorption process is nearly linear, with an R^2^ close to 1. We can also note that a state of equilibrium is reached after 70 to 80 ns, when no more SO_2_ is absorbed by C-111.

### 2.3. Case C

Case C simulations doubled the molecule number of each species used in the previous tests, with the objective to evaluate the possible influence of system size effects on the adsorption results obtained. Then, three simulations at 283 K and 300 K for this particular system were carried out, and the final occupation state was compared vs. the average of case B simulations. The results obtained are listed in Table 2. The computed OR values were, in this case, 0.76 at 283 K and 0.76 at 300 K. These values are compatible with those obtained for case B simulations. Again, in this case, the simulations at 283 K presented slightly higher selectivity.

### 2.4. Case D

**Figure 7:**
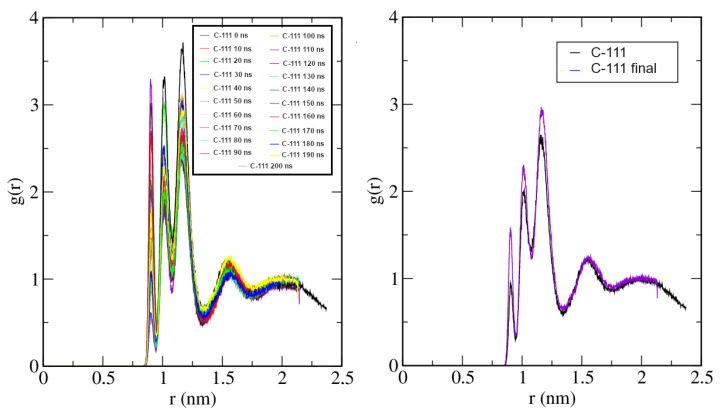
C-111/C-111 radial distribution function at 300 K. **Left**: RDF time evolution along the simulation for 10 ns time stpes. **Right**: Comparison of initial and final states.

The radial distribution functions (RDFs) were analysed in these additional, longer runs, and their time evolution, described. This RDF evolution was analysed for the molecular pairs C-111/C-111, C-111/SO_2_, and SO_2_/SO_2_. The reference point for each molecule was the corresponding centre of mass. Figure 7 shows the time evolution during the simulation of the C-111/C-111 RDF, including a comparison between the initial and final states. The first maximum in this representation is split into three individual peaks. As C-111 is not spherically symmetric, the minimum approach distances depend on their mutual orientations, with three different contact distances. The representation shows a liquid-like structure, and the SO_2_ capture process does not affect the C-111 mutual spatial arrangement.

**Figure 8:**
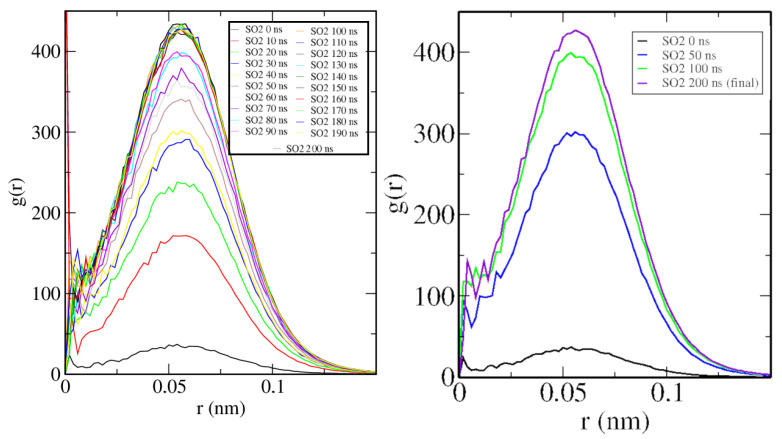
C-111/SO_2_ radial distribution function at 300 K. **Left**: RDF time evolution. **Right**: Comparison of initial and final states, with only two additional intermediate states plotted.

In Figure 8, the time evolution of the C-111/SO_2_ RDF is clearly descriptive of the SO_2_ absorption process by C-111. The single peak at a very short distance represents the location of a SO_2_ molecule inside the C-111 cavity, and the height of this peak grows rapidly as the OR increases.

**Figure 9:**
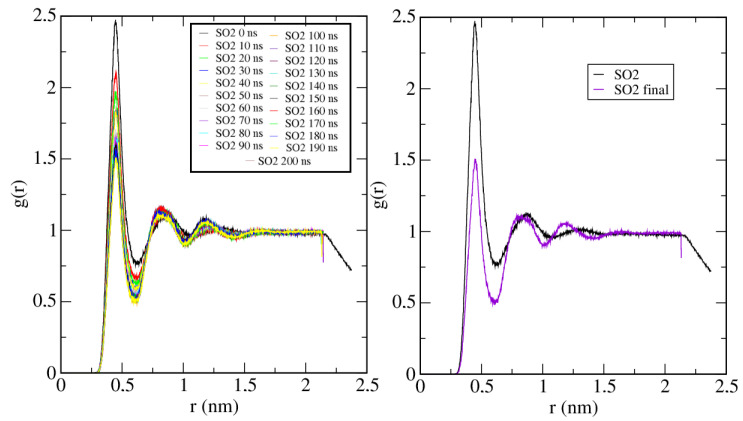
SO_2_/SO_2_ Radial distribution function at 300 K. **Left**: RDF time evolution. **Right**: Comparison of initial and final states.

Finally, in Figure 9, the RDF of SO_2_ molecules is shown, and the trend evidences an increase in intermolecular distances as the simulation advances. As they are being absorbed, a physical barrier is placed between them (C-111), so they cannot approach each other as much as they did initially.

### 2.5. Case E

**Figure 10:**
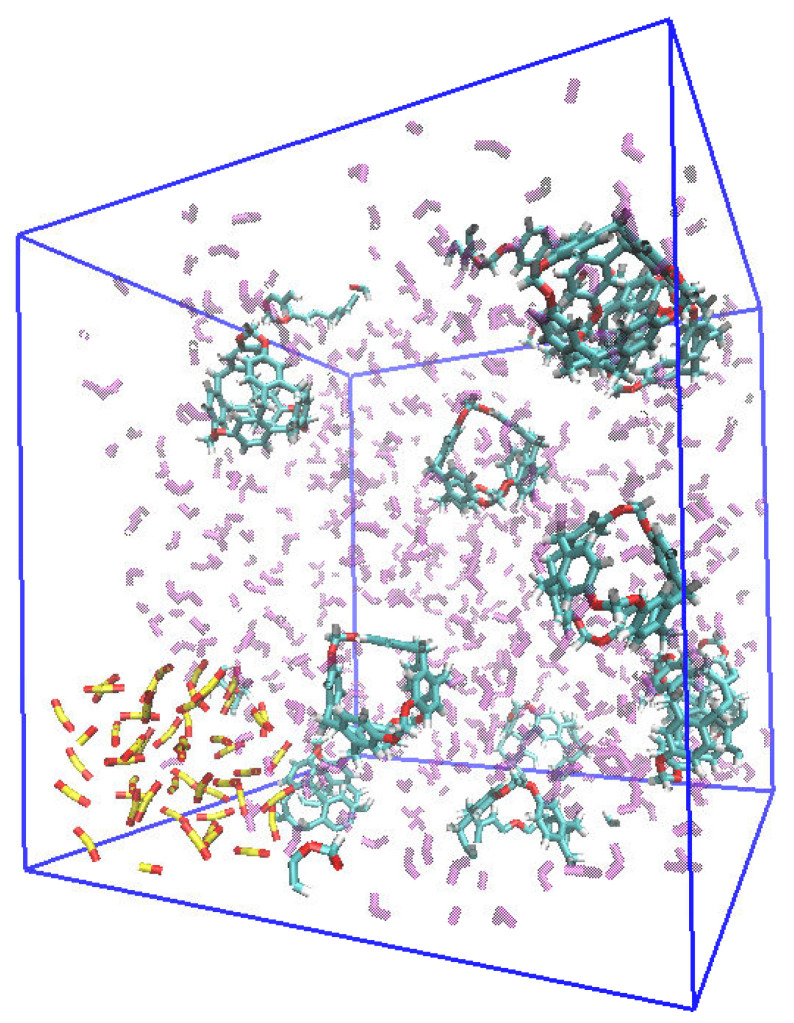
Simulation of the case E system, considering the presence of a SO_2_ bubble.

A common feature observed in cases B and C is that the OR of the C-111 cavities is similar at both temperatures tested, with only slightly higher values in the simulations at 283 K. Despite the increase in system size, this OR does not seem to be affected. In this last case, case E, the system size and proportions were the same as in case D, but all SO_2_ molecules in the system were introduced forming a bubble, instead of being dispersed in the simulation box. This new initial configuration is shown in Figure 10. The motivation for choosing this configuration is twofold. First, this setup approximates the conditions of a flue gas containing SO_2_ being circulated within a C-111-containing DCM bulk. Additionally, it can provide insight into the possible effect of the different diffusion rate of SO_2_ molecules in the system by the effect of a gas–liquid interface in the bubble. The obtained results show that introducing SO_2_ molecules in a bubble does not influence the final OR, with a computed value of 0.75, which is virtually identical to the previous case, case D; thus, the system selectivity is not modified.

### 2.6. Future Approaches

The molecular simulation results obtained allow us to state the difficulty for the SO_2_ molecule to get out of the C-111 cavity with the system setup designed. This indicates a certain degree of interaction between guest and cage molecules, as it was evidenced in our previous study [17]. In the case studied here, the SO_2_ molecule, because of its size or the interaction itself, cannot leave the cavity, contrarily to the behaviour shown by CO_2_. Some more in-depth studies focused on the dynamics of the caption should be carried out to understand the feasibility of a desorption process and the proposal of a renewable method for greenhouse gas capture using this type of PL.

It is clear from the shapes of both guest and host molecules that the movement of the guest molecules is very restricted once trapped by the porous liquid, if compared with the bulk phase. From this perspective, it seems reasonable to assume a significant entropic contribution to the capture process. Additionally, the stability of the guest–host complex observed during the simulations points in the same direction. In order to obtain quantitative evidence, however, free-energy calculations would be advisable. A molecular system of this complexity requires the use of intensive computational resources to take into account a representative number of scenarios. Therefore, this line of research offers many future perspectives. We have also mentioned the short-lived C-111 agglomeration, which seems to increase when these molecules are occupied. A detailed study of this behaviour is also a matter of future interest.

## 3. Computational Methods

The molecular structures used in the simulations performed were obtained from previous literature studies. The C-111 and DCM topologies were used as they were described in our previous work [17]. The C-111 atomic coordinates were retrieved from Joseph et al. [19], who studied the encapsulation of xenon, and the corresponding topology was built using the Automated Topology Builder (ATB) v.3.0 tool [32], where the different charges of the individual atoms are also listed. The molecular model for DCM was also obtained from ATB, according to Stroel et al. [33]. SO_2_ is a widely studied molecule, and in this case, the “MOLID 370654” model was selected among the several available possibilities in the ATB repository.

All molecular dynamics simulations were performed using Gromacs [34,35,36,37,38,39,40,41]. A typical calculation run consisted in 100 ns of simulation with a 1 fs timestep, except when indicated otherwise. An initial energy minimisation operation was performed using the conjugated gradient method to avoid significant molecular overlapping. Electrostatic long-range corrections were handled using the particle mesh Ewald (PME) technique [42], with 1 nm radius, 4th order of calculation, and a Fourier spacing of 0.1 nm. The dispersive van der Waals cut-off radius was set to 1 nm, and long-range dispersive corrections to energy and pressure were applied. The Nose–Hoover [43,44] thermostat was used to keep the temperature constant, with a 2 ps coupling constant, and a Parrinello–Rahman [45,46] barostat was used to set the pressure value, with a 4 ps coupling constant. All simulations were started with random seeds generated by Gromacs, and the final configurations were analysed using VMD open-source software [47,48]. All molecular representations were prepared with VMD and UCSF ChimeraX [49] software.

Five different molecular simulation setups were studied in this work. In all cases, a cubic simulation box with a size of 4.8 nm was used. In the first case, simulation boxes labelled as A contained two different binary mixtures, whose first component was, in both cases, the solvent, DCM, and the second component was either C-111 or SO_2_, at two different temperatures. For the second set of simulations, which were labelled as B, the simulation box consisted of a ternary composition with 7 C-111 molecules, 300 DCM molecules, and 28 SO_2_ molecules. The objective in this second case was to obtain a first evaluation of the SO_2_ encapsulation process by C-111 in a DCM solvent. The third case, which was denoted as C, consisted of simulation boxes whose number of molecules was twice that of case B, thus containing 14 C-111 molecules, 600 DCM molecules, and 56 SO_2_ molecules. The objective of this test was to compare the C-111 occupancy ratio with the previous case, trying to check whether system size effects could be identified at this stage. In the fourth case, which was denoted as D, the same box size as case C was used, but longer production simulation runs were performed (an additional 200 ns). The trajectories obtained were then analysed with a high frame frequency to obtain detailed radial distribution functions (RDFs). The time evolution of the RDFs was obtained by dividing the simulation run in 10 ns periods, at 300 K. For the last case, labelled as E, we introduced a SO_2_ bubble in a simulation box with the composition detailed in case C. The difference from this previous case C was the SO_2_ insertion method. In case C, SO_2_ molecules were inserted at random positions in the solvent, while now, in case E, SO_2_ molecules were inserted concentrated in a spherical bubble placed in one of the corners of the simulation box, trying to mimic an experimental approach. In this last case, an additional 100 ns was considered in the equilibration stage, and the final composition was 12 C-111, 580 DCM, and 56 SO_2_ molecules.

Special care was taken during the initial setup construction to avoid the accidental insertion of a DCM molecule inside a C-111 cavity. The DCM molecular volume is smaller than the maximum cavity volume of C-111, but despite this, DCM cannot spontaneously access the cavity, because the DCM average radius is larger than the cavity threshold. This is the reason why the suspension of C-111 in DCM can be considered a type II porous liquid.

**Figure 11:**
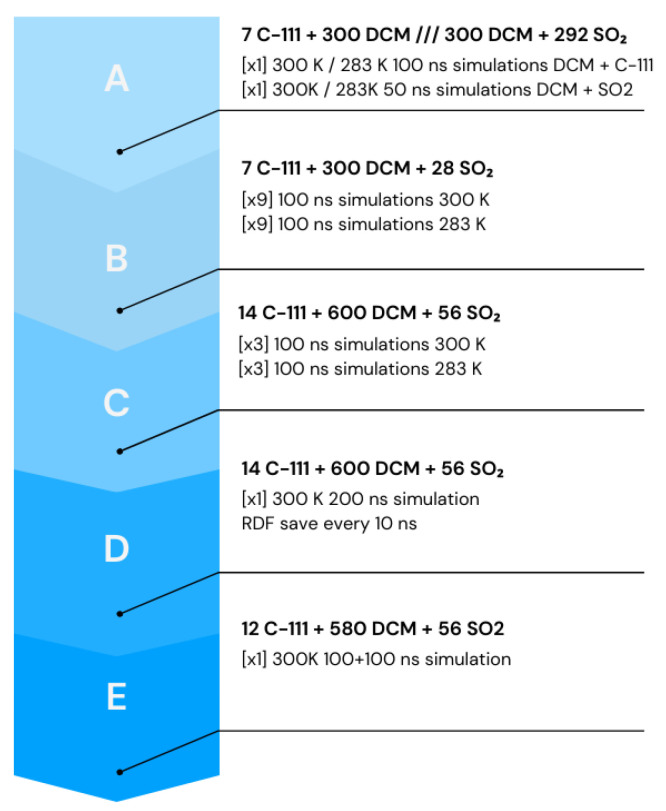
Scheme of the different simulations performed.

**Figure 12:**
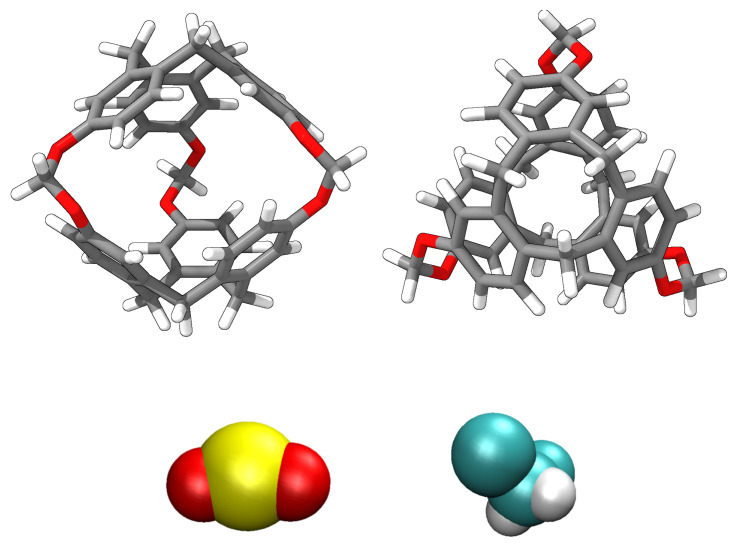
Sketches of the molecular models used for each molecule: C-111 (**top**), SO_2_ (**bottom left**), and DCM (**bottom right**).

Molecular simulations were organised as follows (except for case E, which was specified before): The simulation box was built with the composition explained, by inserting first C-111 molecules and then the DCM solvent molecules, preventing the accidental insertion of DCM inside C-111 cavities. Finally, the SO_2_ molecules were also inserted at random, and in this case, the inner C-111 porosity was not blocked. After the energy minimisation step, NpT molecular dynamics were performed at 0.1 MPa and temperatures of 300 K and 283 K. Nine different replica boxes at both temperatures for case B were built, and three boxes for each temperature for case C. In cases D and E, only one simulation run was carried out. The organisation of the different simulations is presented schematically in Figure 11. A representation of the used molecular models is presented in Figure 12. Every cavity occupation for case B was recorded through the simulations to compare the evolution of each species under the different conditions explored. In C case only the final occupation was retrieved.

## 4. Conclusions

This study is a contribution to the theoretical modelling and characterization of porous liquids and postulates a potential method for safe and easy SO_2_ separation and recovery through adsorption. The simulations performed show that SO_2_ adsorption is highly favoured inside C-111 cavities in a DCM solvent. SO_2_ tended to occupy almost all the empty accessible pores during the simulation. Temperature has a slight influence on the occupation of the pores in the range tested, and the adsorption process is faster at higher temperatures, as expected. There is no noticeable change in the process when the initial number of molecules is doubled, suggesting a scalable process. The more experimental-like approach of introducing a SO_2_ bubble in the box has also no influence on SO_2_ pore occupation. Multiple-SO_2_ occupancy inside C-111 cavities was not observed and can be discarded as a possibility. The interaction between SO_2_ and the C-111 cavity favours the capture process. Mutual interactions between C-111 molecules in DCM solution are weak, although not negligible, but harmful agglomeration effects were not noticed.

These results point out towards porous liquids as a potential alternative in the development of innovative strategies towards greenhouse gas capture and separation, due to their intrinsic porosity and the high selectivity they present towards some of these gases. The possibility of using them as a renewable option for the capture of this gases opens a path towards interesting applications in the energy and global change fields of knowledge.

## Figures and Tables

**Table 1:** Case B: C-111 cavity occupation by SO_2_ molecules.

**300 K**	
**Simulations**	**Occupation (Out of 7)**
REP1	6
REP2	5
REP3	6
REP4	6
REP5	7
REP6	7
REP7	5
REP8	4
REP9	7
Average	5.9±1.0
**283 K**	
**Simulations**	**Occupation (Out of 7)**
REP1	5
REP2	5
REP3	6
REP4	7
REP5	6
REP6	6
REP7	7
REP8	7
REP9	6
Average	6.1±0.8

**Table 2:** Case C cavity occupation.

**300 K**	
**Simulations**	**Occupation (Out of 14)**
REP1	11
REP2	10
REP3	10
Average	10.3
**283 K**	
**Simulations**	**Occupation (Out of 14)**
REP1	11
REP2	11
REP3	10
Average	10.6

## Data Availability

The original contributions presented in the study are included in the article, and further inquiries can be directed to the corresponding author.

## Abbreviations

The following abbreviations are used throughout the manuscript:

ATB	Automatic Topology Builder
DCM	dichloromethane
C-111	cryptophane-111
MOF	metal organic framework
OR	occupancy ratio
PDB	Protein Data Bank
PL	porous liquid
